# Assessing the combined impact of fatty liver-induced TGF-β1 and LPS-activated macrophages in fibrosis through a novel 3D serial section methodology

**DOI:** 10.1038/s41598-024-60845-6

**Published:** 2024-05-18

**Authors:** Shiori Ishiyama, Manabu Hayatsu, Taku Toriumi, Hiromasa Tsuda, Keisuke Watanabe, Hirotake Kasai, Satoshi Kishigami, Kazuki Mochizuki, Yoshikazu Mikami

**Affiliations:** 1 Department of Integrated Applied Life Science, Integrated Graduate School of Medicine, Engineering, and Agricultural Sciences, University of Yamanashi, Yamanashi, Japan; 2 Division of Microscopic Anatomy, Graduate School of Medical and Dental Sciences, Niigata University, Niigata, Japan; 3 Department of Anatomy, The Nippon Dental University School of Life Dentistry at Niigata, Niigata, Japan; 4 Department of Biochemistry, Nihon University School of Dentistry, Tokyo, Japan; 5 Division of Gross Anatomy and Morphogenesis, Graduate School of Medical and Dental Sciences, Niigata University, Niigata, Japan; 6 Department of Microbiology, Faculty of Medicine, Graduate Faculty of Interdisciplinary Research, University of Yamanashi, Yamanashi, Japan; 7 Center for Advanced Assisted Reproductive Technologies, University of Yamanashi, Yamanashi, Japan

**Keywords:** Mechanisms of disease, Molecular medicine

## Abstract

Non-alcoholic steatohepatitis (NASH), caused by fat buildup, can lead to liver inflammation and damage. Elucidation of the spatial distribution of fibrotic tissue in the fatty liver in NASH can be immensely useful to understand its pathogenesis. Thus, we developed a novel serial section-3D (SS3D) technique that combines high-resolution image acquisition with 3D construction software, which enabled highly detailed analysis of the mouse liver and extraction and quantification of stained tissues. Moreover, we studied the underexplored mechanism of fibrosis progression in the fatty liver in NASH by subjecting the mice to a high-fat diet (HFD), followed by lipopolysaccharide (LPS) administration. The HFD/LPS (+) group showed extensive fibrosis compared with control; additionally, the area of these fibrotic regions in the HFD/LPS (+) group was almost double that of control using our SS3D technique. LPS administration led to an increase in *Tnfα and Il1β* mRNA expression and the number of macrophages in the liver. On the other hand, transforming growth factor-β1 (*Tgfβ1*) mRNA increased in HFD group compared to that of control group without LPS-administration. In addition, COL1A1 levels increased in hepatic stellate cell (HSC)-like XL-2 cells when treated with recombinant TGF-β1, which attenuated with recombinant latency-associated protein (rLAP). This attenuation was rescued with LPS-activated macrophages. Therefore, we demonstrated that fatty liver produced “latent-form” of TGF-β1, which activated by macrophages via inflammatory cytokines such as TNFα and IL1β, resulting in activation of HSCs leading to the production of COL1A1. Moreover, we established the effectiveness of our SS3D technique in creating 3D images of fibrotic tissue, which can be used to study other diseases as well.

## Introduction

Non-alcoholic fatty liver disease (NAFLD) is characterized by fat accumulation in the liver occurring independently of habitual or chronic alcohol consumption^1^. NAFLD manifests in two forms: simple steatosis, which is only lipid accumulation in hepatocytes, and non-alcoholic steatohepatitis (NASH)^2^. NASH, the more severe form of NAFLD, involves necrosis, inflammation of liver tissues, and fibrosis and accounts for 10–20% of NAFLD cases^3^. Progression to liver cirrhosis or liver cancer occurs within 10 years in 20–30% of NASH cases^4^.

The multiple parallel hit theory has been put forth to demonstrate the pathogenesis of NASH^5^. This theory posits that the development of the disease involves several pathological processes occurring simultaneously. Instead of a single underlying cause, multiple factors are considered to contribute to the progression of NASH. These processes interact and exacerbate each other, leading to the characteristic features of the disease, including hepatic steatosis, inflammation, and fibrosis. For example, factors such as increased triglyceride levels in the liver and insulin resistance, often due to obesity and lipid accumulation, lead to simple steatosis. In this state, hepatocytes damaged by excessive lipid accumulation release damage-associated molecular patterns (DAMPs), including nucleic acids, cytokines, and lipid-based molecules, which trigger non-infectious chronic hepatic inflammation through the recruitment of macrophages^6–8^. The other factors include the production of inflammatory cytokines, induced by oxidative stress and pathogen-associated molecular patterns (PAMPs) such as lipopolysaccharides (LPS)^9,10^. LPS activates macrophages, inducing them to produce inflammatory cytokines such as tumor necrosis factor-α (TNF-α) and interleukin-1 (IL-1). Inflammatory cytokines activate hepatic stellate cells (HSCs) in the liver. Analyses using animal models of liver fibrosis indicate that HSCs are the primary cells responsible for fibrosis production in the liver^6,8, 11^. Under normal conditions, HSCs are quiescent, store vitamin A, and regulate sinusoidal microcirculation by contracting and relaxing in response to various vasoactive mediators^12^. However, when activated by inflammatory cytokines, HSCs transform into myofibroblasts, which contribute to the formation of fibrous tissues by synthesizing and secreting extracellular matrix molecules such as collagen^12^. Activated HSCs have also been reported to produce transforming growth factor-β1 (TGF-β1), which is known as a cytokine that induces fibrosis and acts in an autocrine manner^13^. TGF-β1 is produced by various types of cells, including M2-type macrophages as well as HSCs^14^. The production of cytokines such as TNF-α, IL-1, and TGF-β1 is promoted by signals downstream of DAMPs and PAMPs. However, DAMPs and PAMPs activate various signals through specific receptors that interact intricately with each other^15–18^. Therefore, their interplay is complex and multifaceted, and the mechanism behind DAMP/PAMP-induced fibrosis progression in fatty liver is not fully understood. Thus, in this study, ICR mice were subjected to a high-fat diet (HFD) as the first hit, followed by LPS as the second hit. The livers of these mice were then analyzed to shed light on the mechanisms of NASH pathogenesis.

Various cell types, including hepatocytes, HSCs, and macrophages, play crucial roles in the development of NASH^19–21^. Additionally, the liver is a complex organ composed of vessels, lymphatic channels, bile ducts, and specialized structures such as Disse's space. Understanding the spatial distribution of these cells and structures, along with fibrotic tissue in three dimensions (3D), is vital for elucidating the mechanisms of NASH pathogenesis. Recently, 3D analytical techniques employing tissue transparency technology and light-sheet fluorescence microscopy have gained significant attention^22,23^. However, these techniques require fluorescent labeling of target cells and structures. Conversely, conventional 3D analysis, which integrates 2D images of tissue sections, demands considerable time and effort for stitching and aligning images.

In this study, we introduce a novel serial section-3D (SS3D) construction method. This approach combines high-resolution image acquisition using a virtual slide scanner with 3D construction software, incorporating a unique computational deep learning program. This enables efficient, high-throughput 3D analysis of the mouse liver, ranging from whole organ imaging down to single-cell resolution. Additionally, this method allows for the extraction and quantification of stained structures using various staining techniques. The effectiveness and utility of the SS3D method were also evaluated.

## Materials and methods

### Animals

To induce obesity and NAFLD, 5-week-old ICR mice purchased from Japan SLC (Shizuoka, Japan) were bred inhouse in cages (five per cage). The mice were allowed ad libitum access to high-fat diet (HFD) (Oriental Yeast Co., Ltd., Nagahama, Japan) or normal diet (ND) and water; they were reared at a temperature of 23 °C ± 2 °C and humidity of 50% ± 10%, with a 12-/12-h reversed dark–light cycle (2000–0800 h) for 12 weeks as previously reported^24,25^. It has been shown that inflammatory cytokine concentrations increase in the liver within 2 h of LPS administration in ICR mice^26^. However, the formation of fibrotic tissue in the liver under the influence of these cytokines takes more time because proteins such as collagen must be synthesized through various signaling pathways. On the contrary, the pain of experimental animals must be minimized to the greatest extent possible. Therefore, in this study, the LPS administration time was set to 48 h as the minimum time for the formation of notable fibrotic tissue. Regarding the concentration of LPS, 1–10 mg/kg is generally used to investigate its inflammatory effect in vivo^26,27^, but it varies depending on the experimental conditions. Therefore, 2.5, 5, and 10 mg/kg were considered in this study. After feeding HFD for 12 weeks, mice were randomly divided into two groups of similar body weight: ND and HFD group, and mice were injected with LPS (124‐05151; WAKO Pure Chemical Industries, Osaka, Japan) at a dose of 2.5 (each n = 6), 5 (each n = 6) and 10 (each n = 7) mg/kg BW or an equivalent volume of the vehicle control (phosphate-buffered saline; PBS, n = 6) once intraperitoneally. Dissections were performed 48 h after the injection. The liver and mesenteric fatty tissue samples for mRNA analysis were collected immediately snap-frozen and stored at − 80 °C until analysis. All protocols with mice were performed under the approval of the Ethics Committee of the University of Yamanashi (approval number A2-25), and conducted in accordance with the ARRIVE guidelines and the standards of humane animal care by the criteria outlined in the “Guide for the Care and Use of Laboratory Animals”.

### Cells

Primary human macrophages from blood monocytes isolated from healthy donors and XL-2 cell line were obtained from the Riken Cell Bank (Ibaragi, Japan) and Sigma-Aldrich Co. (St. Louis, MO, USA), respectively. Antibiotics and cell culture medium were purchased from Gibco (Grand Island, NY, USA). Fetal bovine serum (FBS) was purchased from Japan Bioserum Co., Ltd. (Tokyo, Japan). Recombinant (r) human TGF-β1 (active form) and latency associated peptide (TGF-β1) (LAP) protein were purchased from R&D Systems (Minneapolis, MT, USA) and Sigma-Aldrich Co., respectively. Sigma Chemical Co. (St. Louis, MO, USA). Cells (5 × 10^4^) were seeded on 35 × 10 mm polystyrene Petri dishes with physical surface treatment (Greiner Bio-One International GmbH, Kremsmünster, Austria) and maintained in the standard medium (Dulbecco’s modified Eagle’s medium (DMEM) containing 10% FBS and 1% penicillin–streptomycin) at 37 °C in a humidified atmosphere containing 5% CO_2_.

### Sectioning

Serial sections (5 µm thickness) were cut from paraffin-embedded samples using a sliding microtome (ROM-380, Yamato Koki Co. Ltd., Saitama, Japan) and mounted onto glass slides (CREST, Matsunami Glass, Osaka, Japan), as shown in Suppl. Data 1. Hundreds of serial paraffin sections were mounted on glass slides by repeating this sectioning procedure. Category 1 of the sections shown in Suppl. Data 1 was used for azan staining to observe the liver. Categories 2–5 of sections were used for immunohistochemical staining.

### Azan staining

Azan staining was performed as described previously^28^, for category 1 sections shown in Suppl. Data 1. Briefly, paraffin-embedded sections were deparaffinized with xylene (two times) and rehydrated using a series of graded ethanol (100%, 95%, 90%, 80%, 70% of ethanol) at 25 ℃ for 10 min each. After washing with running tap water for 5 min, the sections were stained with Azan solution. Then, the sections were dehydrated using 95% ethanol (two times), 100% ethanol (two times) each for a few seconds, and xylene (three times) at 25 ℃ for 1 h each. Dehydrated sections were mounted with mounting medium and dried at 25 ℃.

### Immunohistochemical staining

Immunohistochemical staining was performed as described previously^29^, in category 2 and 4 of the sections shown in Suppl. Data 1 that shows the localization of macrophages. Categories 3 and 5 of sections were used to obtain immunohistochemical images of the other cells (data not shown). Similar to the Azan staining method, paraffin-embedded sections were deparaffinized with xylene and rehydrated using a series of graded ethanol. The sections were soaked in 10 mM sodium citrate buffer (pH 6.0), and antigenicity was retrieved by preheating at 121 °C for 20 min in an autoclave. Following washing in PBS at 25 ℃ for 3 min, and endogenous peroxidase activation was blocked by incubation with 0.3% hydrogen peroxide solution at 25 ℃ for 10 min. After washing in PBS at 25 ℃ for 3 min, and non-specific binding of the antibodies was blocked by a 30-min incubation at 25 ℃ using solution containing 4% Block Ace (Yukijirushi, Hokkaido, Japan). After washing in PBS at 25 ℃ for 3 min, the sections were incubated with diluted primary antibodies (F4/80: #30325; Col1A: #72026, Cell Signaling Technology, Danvers, MA, USA) in 1% BSA in a humidified chamber at 4 °C overnight. Following washing in PBS-T (PBS with 0.05% Tween 20) at 25 ℃ for 5 min, non-specific binding of the antibodies was blocked by a 30-min incubation at 4 °C in a humidified chamber using 10% normal goat serum from the species of the secondary antibody (Histofine SAB-PO (R) kit, Nichirei, Tokyo, Japan). The sections were then incubated with a goat anti-rabbit IgG-HRP secondary antibody (Histostar, MBL, Nagoya, Japan) in a humidified chamber at 4 °C for 30 min. After washing twice with PBS-T at 25 ℃ for 5 min, HRP activity was visualized by incubation in 0.05% 3, 3-diaminobenzidine solution (Histofine SAB-DAB kit, Nichirei, Tokyo, Japan). Sections were counterstained with Mayer’s hematoxylin at 25 ℃ for 15 min, washed in running tap water for 15 min, dehydrated with 95% ethanol (two times), 100% ethanol (two times) each for a few seconds, and xylene (three times) for 1 h each. Dehydrated sections were mounted with a mounting medium and dried at 25 ℃.

### SS3D reconstruction

Image acquisition was performed automatically using the virtual slide system (Nanozoomer S210, Hamamatsu photonics K.K., Shizuoka, Japan). The serial section images of blood vessels and fibrous tissues were obtained from Azan staining images. In addition, macrophages selectively stained using immunohistochemical techniques were obtained. For the 3D reconstruction of fibrous tissues, vessels, and macrophages from 2D images, the digital images were loaded into the proprietary modified software based on Amira Software (Thermo Fisher Scientific Inc, MA, USA). Adjustment of the positions and boundary inclinations of serial section images was performed using the tool in this software. In brief, we applied the process as follows: (1) Align Slices, (2) Extract Subvolume, (3) Interactive Thresholding, (4) Binary Smoothing, (5) Generate Surface, and (6) Surface View.

### Quantitative real-time RT-PCR (qRT-PCR)

Extraction of total RNA from liver or cultured cells and qRT-PCR were performed as described previously^30^. In brief, cDNA was produced using SuperScript III reverse transcriptase (Thermo Fisher Scientific Inc.), following the manufacturer’s instructions. The LightCycler 480 Universal Probes Master and LightCycler System (Roche Diagnostics K.K.) was used for qRT-PCR. The mRNA primer sequences are shown in Suppl. data 2. The mRNA levels were detected relative to an internal control gene (*Gapdh* following the delta-delta) Ct (threshold cycles) method.

### Flow cytometry

Flow cytometry analysis was performed as previously described^31^. Briefly, blood cell populations in the liver tissue of mice receiving intraperitoneal administration of LPS were analyzed using flow cytometry. Liver samples were minced and incubated with 10 U/mL Collagenase (CLS4 Type IV, #LS004186, Worthington Biochemical Corporation, Lakewood, NJ) and 50 U/mL DNase I (#2270A, Takara Bio, Shiga Japan) in PBS for 30 min at 37 °C. Single-cell suspensions were prepared by filtration of collagenase-treated tissue through a cell strainer, followed by erythrocyte disruption with ACK (Ammonium-Chloride-Potassium) lysing buffer and washing with 10× volume of PBS buffer. The samples were blocked with 10% FCS-containing FACS buffer for 20 min on ice and incubated with the following direct fluorescent dye-labeled antibodies for 30 min on ice: anti-CD11b FITC (#101206); anti-Gr-1/Ly6C APC (#143905) by Biolegend, Inc (San Diego, CA).

### Co-culture experiments

Co-culture were performed in 6-well plates using a transwell culture system (Becton, Dickinson and Company, Franklin Lakes, NJ, USA) as described previousy^32^ The macrophages were plated onto the filters (pore size, 1 μm) forming the bottom of the upper chambers at a density of 2 × 10^5^ cells per chamber, and the XL-2 cells were plated into the lower wells at a density of 2 × 10^5^ cells per well. The upper chambers were then inserted into the lower wells, and the cells were cultured for 48 h in the standard medium supplemented with or without rTGF-β1 (10 ng/mL), rLAP (100 ng/mL), and LPS (100 ng/mL) at 37 °C under a humidified atmosphere containing 5% CO_2_.

### Statistical analyses

Data are expressed as the mean ± SEM (n = 4–6). For all calculations, two tailed tests were used. To assess the statistical significance of treatment effects, Steel–Dwass or Tukey’s test was employed. The former was used for data not following a normal distribution (Fig. 5) and the latter for data following a normal distribution (Fig. 7). For Steel–Dwass test, the observations across all treatment groups and the control group were ranked. Subsequently, the mean ranks between the treatment groups and the control group were compared. All statistical analyses were performed using the software MEPHAS (Research Institute for Microbial Diseases, Osaka University, Osaka, Japan).

## Results

### Effects of LPS administration on the survival rate in a murine fatty liver model

**Figure 1:**
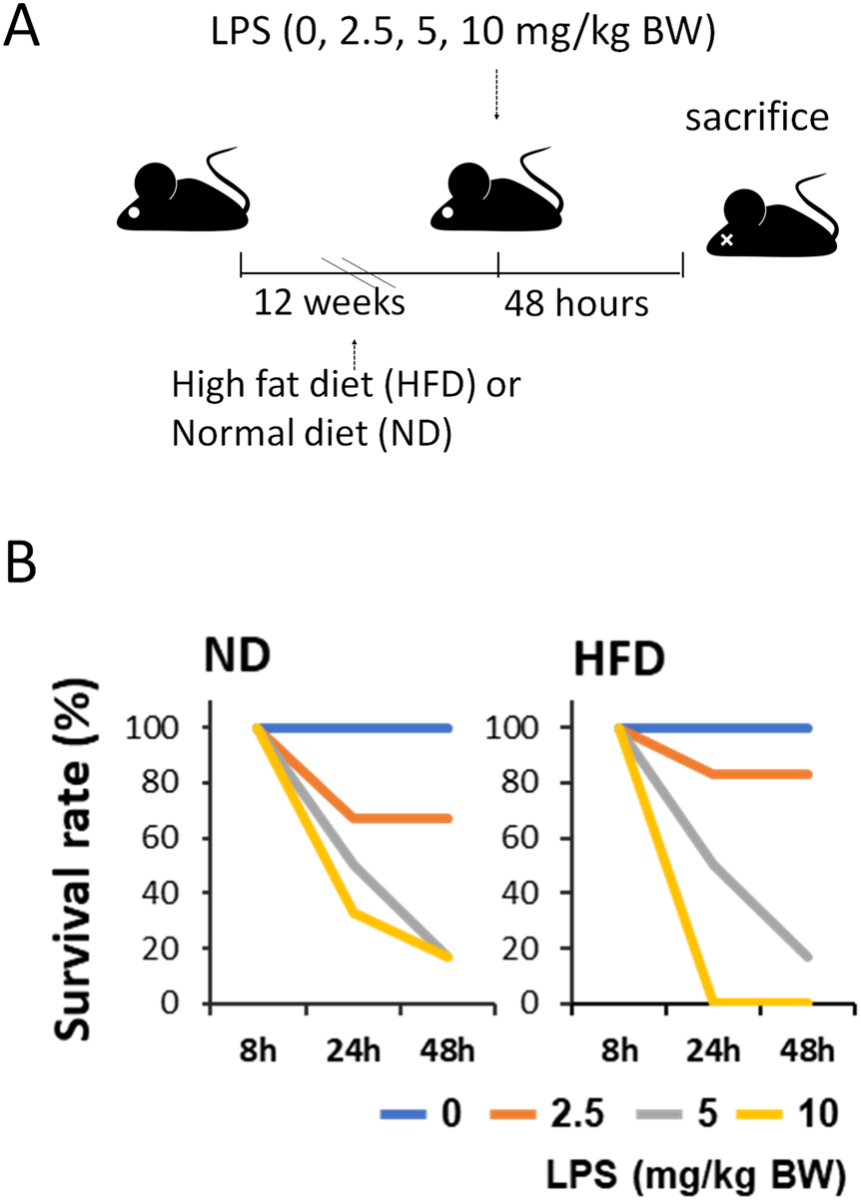
(**A**) Experimental procedures. After feeding high-fat diet (HFD) or normal diet (ND) for 12 weeks, mice were injected with lipopolysaccharides (LPS) at the indicated concentrations. Dissections were performed 48 h after the injection. (**B**) Survival rates of the mice. Survival rates of the mice in four groups' post-treatment period were calculated.

ICR mice were subjected to HFD for 12 weeks to induce fatty liver. Subsequently, LPS was administered, and analyses were conducted 48 h later (Fig. 1A). The survival rates following the administration of 2.5, 5, or 10 mg of LPS to the mice were recorded (Fig. 1B). In control mice fed a normal diet (ND), the survival rates 48 h after receiving 5 and 10 mg of LPS were 16.7% (1 out of 6). In the HFD group treated with 5 mg LPS, the survival rate 48 h later was also 16.7%, mirroring the survival rate of the ND group. However, in the group that received 10 mg LPS, all mice succumbed within 24 h of administration. Following the administration of 2.5 mg LPS, the survival rates after 48 h were 83.3% (5 out of 6) in the HFD group and 66.7% (4 out of 6) in the ND group. As the survival rates with administration of 5 and 10 mg of LPS were below 50% in both the HFD and control groups, 2.5 mg LPS was selected for further experiments.

### Histochemical analysis of the effects of LPS administration on fibrosis in the fatty liver

**Figure 2:**
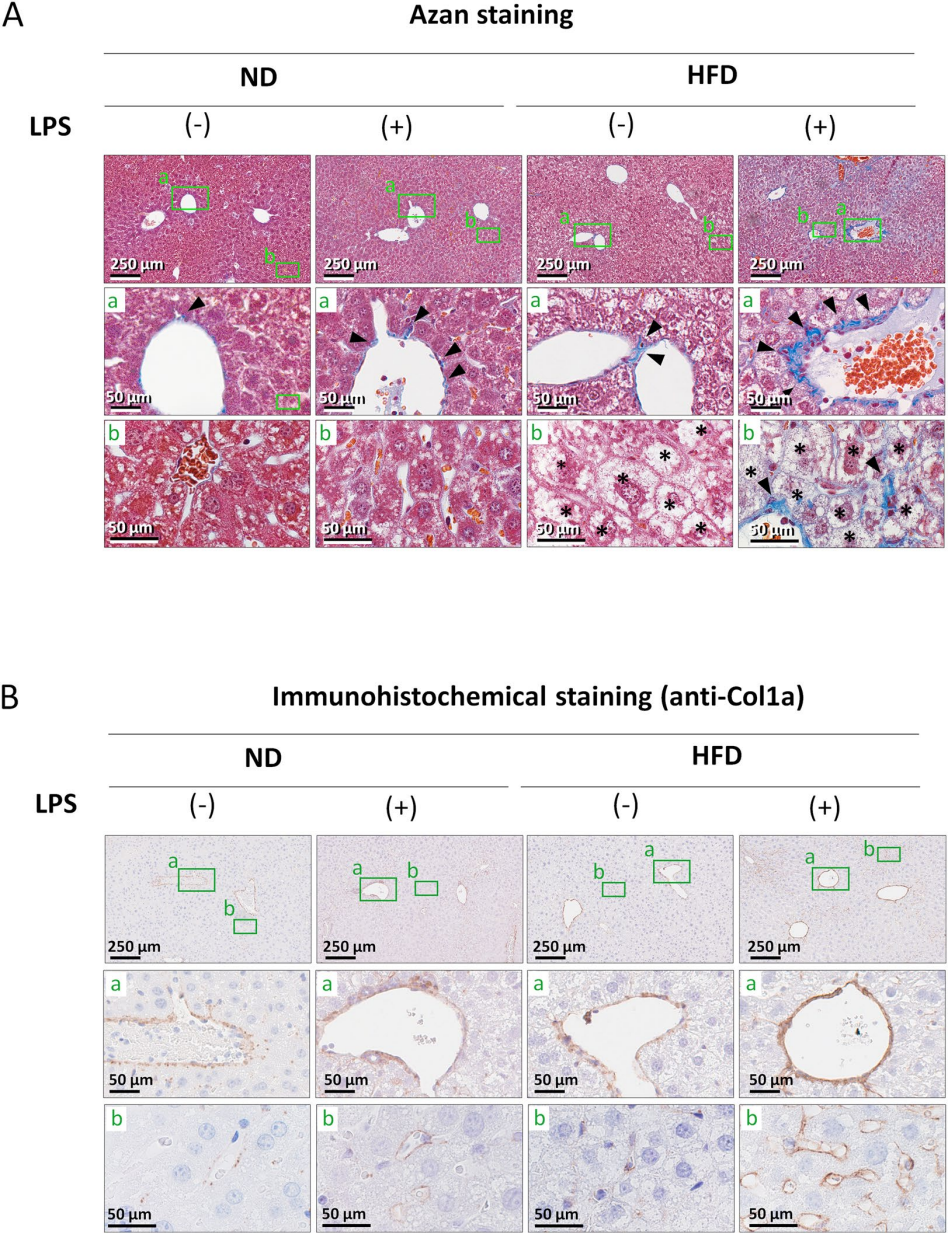
(**A**) Azan staining of liver sections. Liver sections were prepared from mice treated as shown in Fig. 1A. The fibrous connective tissues in each specimen were stained blue (arrow heads). Asterisks indicate ballooning hepatocytes. HFD, high-fat diet; ND, normal diet. (**B**) Immunohistochemical staining of liver sections. Liver sections were prepared as indicated in (**A**), and then immunohistochemical staining using anti-collagen type 1a (Col1a) antibody was performed. 3,3′-Diaminobenzidine (DAB) was used to detect the Col1a signals (brown: arrow heads). Nuclei stained with hematoxylin (blue).

Following the administration of LPS (2.5 mg), the livers from the treated mice (as depicted in Fig. 1A) were examined using histochemical staining methods. Representative azan-stained images for each group are presented in Fig. 2A. Compared to the ND group, the HFD group exhibited increased ballooning degeneration, a characteristic of fatty liver, irrespective of LPS administration, leading to fatty liver. Fibrotic regions (stained blue) were predominantly observed around the perivascular areas of large blood vessels in all groups (Fig. 2A, upper and middle panels). Notably, the number of fibrotic regions was more pronounced in the HFD/LPS(+) group compared with the other three groups. Extensive fibrosis was evident in the perivascular areas of sinusoidal capillaries in the HFD/LPS(+) group (Fig. 2A, lower panels). However, the extent of fibrosis in the other three groups showed no notable differences. Additionally, immunohistochemical staining with an anti-collagen type I α1 (COL1A1) antibody was conducted to corroborate the azan staining results. COL1A1, a major component of fibrotic tissue, yielded results similar to those observed with azan staining (Fig. 2B). In particular, the fibrosis observed in the peri-sinusoidal capillaries, especially prominent in the HFD/LPS(+) group, was consistent with the azan staining findings (Fig. 2B, lower panels).

### Application of SS3D analysis of liver fibrosis

**Figure 3:**
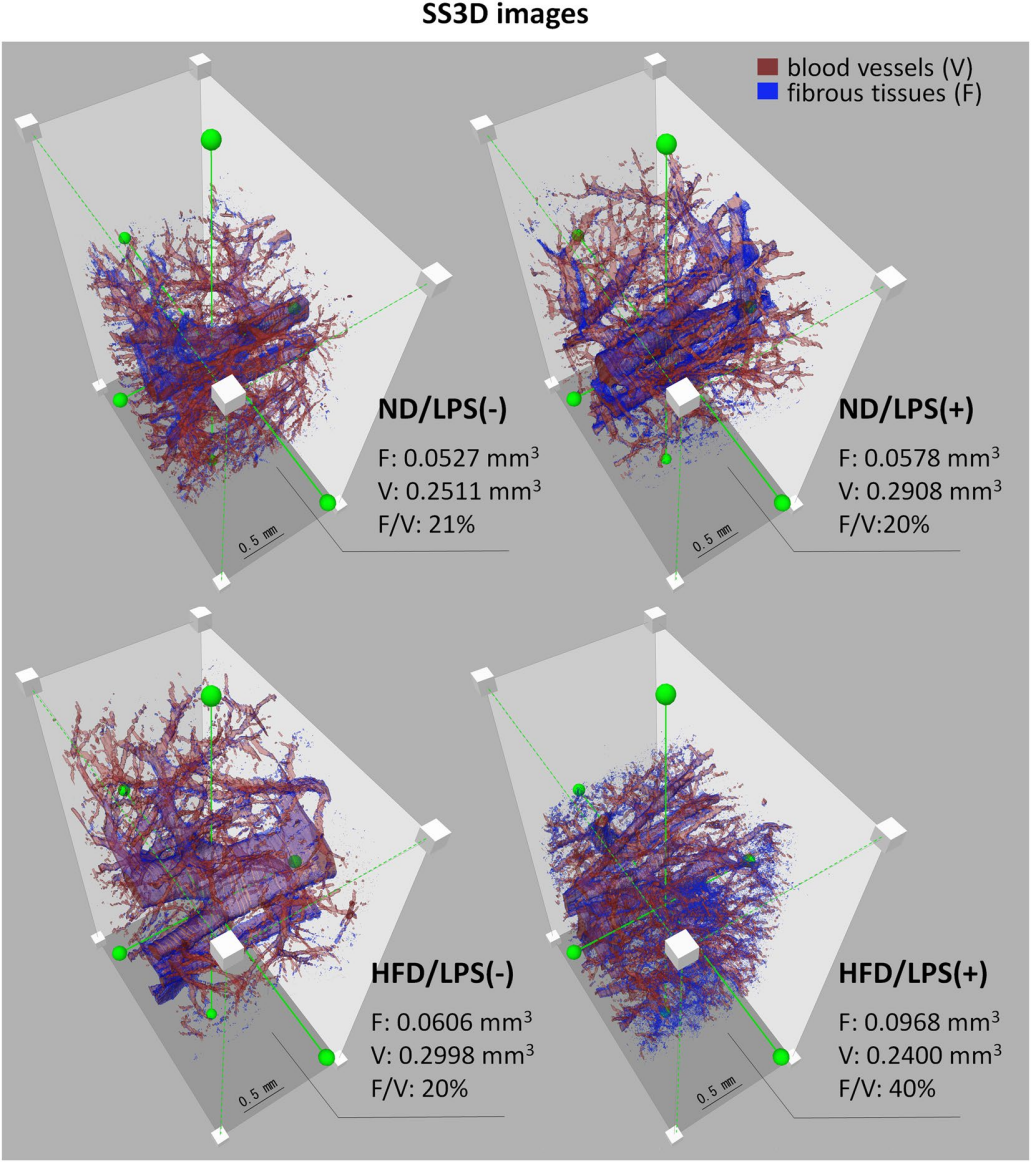
Serial section-3-dimensional (SS3D) structural analysis of the murine livers. Blood vessel (red), and fibrotic tissues (blue) were detected and superimposed, demonstrating that fibrotic tissues increased in HFD/LPS(+) compared to those of other three groups. The values in the lower right corner indicate the surface volume of blood vessel, the total volume of fibrous tissue, and their respective values, respectively. Other details of the experiment are included in Materials and Methods. HFD, high-fat diet; ND, normal diet; LPS, lipopolysaccharides; F, fibrous tissues (blue); V, blood vessel (red).

**Figure 4:**
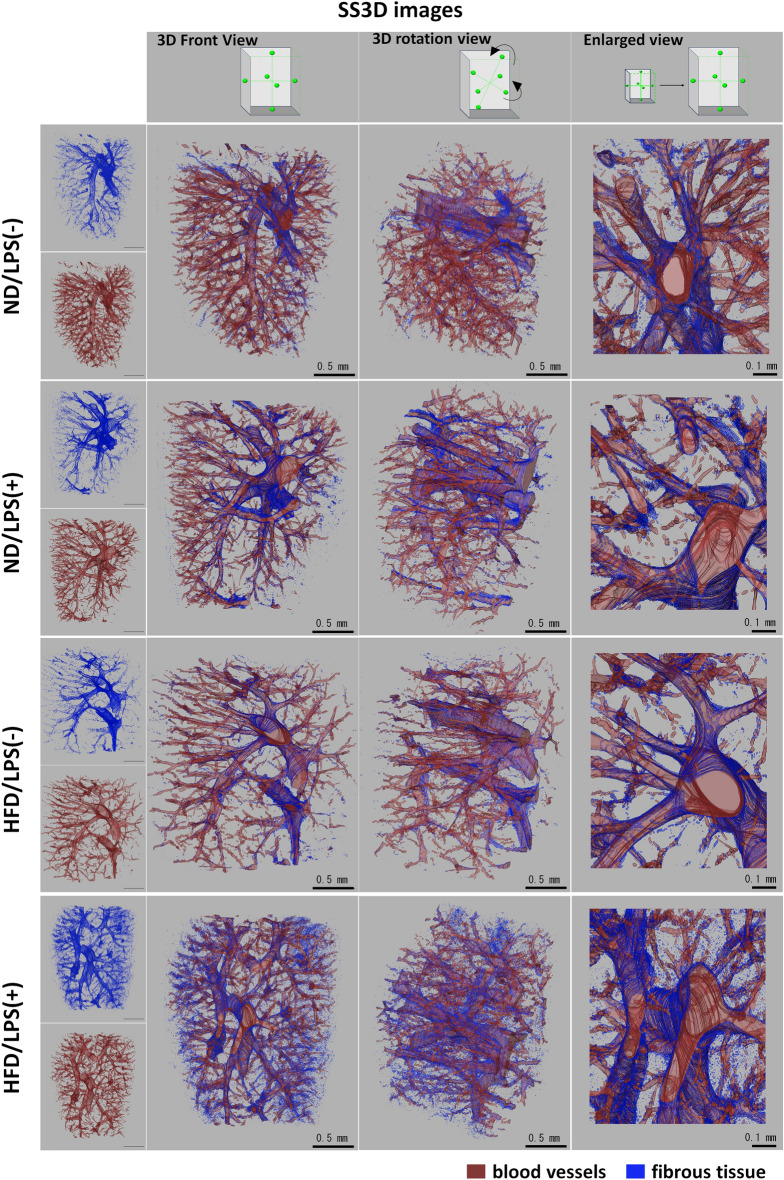
Serial section-3-dimensional (SS3D) images of the murine livers from several angles. The schematic in the upper panels indicates the respective angles. Similar to Fig. 3, increases in fibrous tissues were detected in HFD/LPS(+) compared to those of other three groups. Other details of the experiment are included in Materials and Methods. HFD, high-fat diet; ND, normal diet; LPS, lipopolysaccharides; F, fibrous tissues (blue); V, blood vessel (red).

SS3D analysis was utilized to examine sections of the murine liver. Blood vessels and fibrotic tissues were isolated from azan-stained images, followed by 3D reconstruction (Figs. 3 and 4). Given that the predominant formation of fibrotic tissues was observed in the perivascular regions (Fig. 2), the fibrosis was quantified by measuring the surface volume of blood vessels and the total volume of fibrotic tissue. The corresponding values and their relative proportions are depicted in Fig. 3. Figure 4 includes snapshots and magnified images captured from various angles. In the 3D images of all groups, a pattern of numerous blood vessels branching from larger ones and progressively narrowing was observed. However, no significant differences in vascular status were noted among the four groups. Consistent with Fig. 2, fibrotic tissue primarily surrounded the blood vessels in all groups. In the HFD/LPS(+) group, the fibrotic tissue around the sinusoidal capillaries, as shown in Fig. 2, appeared in the liver parenchyma as dots, some of which aligned in a linear series. The proportional area of fibrotic tissue was approximately double in the HFD/LPS(+) group compared with the other three groups (approximately 40% versus 20%, respectively). Yet, when comparing the three groups excluding the HFD/LPS(+) group, there were no significant differences in the presence of fibrotic tissue.

### Effects of LPS administration on inflammation-related gene expression in the murine model of fatty liver

**Figure 5:**
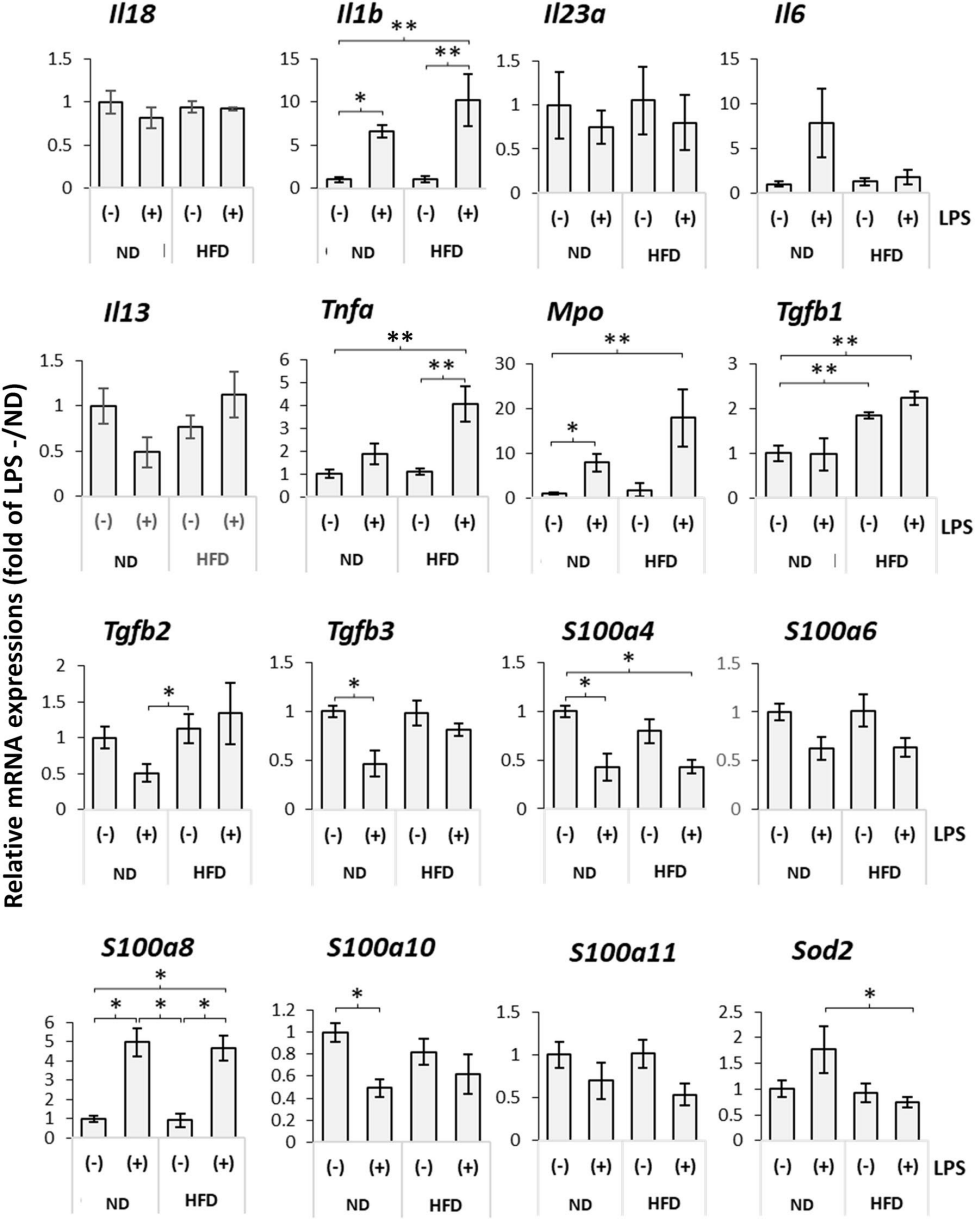
Expression levels of the inflammation-related genes. Real-time RT-PCR was performed to investigate the mRNA expression level of the indicated inflammation-related genes in liver tissues of the mice treated as shown in Fig. 1A. Quantitative data were presented as the mean ± SEM [ND/LPS(−): n = 6; ND/LPS(+): n = 4; HDF/LPS(−): n = 6, HFD/LPS(+): n = 5]]. Single and double asterisks indicate statistically significant (*P* < 0.05) and marginally significant (0.05 < *P* < 0.1) differences between the samples indicated with bars, respectively. No statistically significant differences were observed among the other samples. Interleukin-5, interleukin-17A, interleukin-21, interleukin-22, and interferon regulatory factor-1 were also analyzed, and their mRNA expression was not detectable in any of the groups. HFD, high-fat diet; ND, normal diet; LPS, lipopolysaccharides; Il18, interleukin-18; *Il1b*, interleukin-1 beta; *Il23a*, interleukin-23 alpha; *Il6*, interleukin-6; *Il13*, interleukin-13; *Tnfa*, tumor necrosis factor alpha; *Mpo*, myeloperoxidase; *Tgfb1*, transforming growth factor beta 1; *Tgfb2*, transforming growth factor beta 2; *Tgfb3*, transforming growth factor beta 3; *S100a4*, S100 calcium binding protein a4; *S100a6*, S100 calcium binding protein a6; *S100a8*, S100 calcium binding protein a8; *S100a10*, S100 calcium binding protein a10; *S100a11*, S100 calcium binding protein a11; *Sod2*, superoxide dismutase 2.

Inflammation-related genes are known to play significant roles in fibrosis associated with fatty liver disease. This study analyzed changes in the expression of inflammation-related factors in treated mice, as illustrated in Fig. 1A (refer to Fig. 5 for results). Among the genes examined, the mRNA expression of *Tnfα*, *Il1b*, *Mpo*, and *S100a8* increased following LPS administration. Notably, the LPS-induced increase in *Tnfα*, *Il1b*, and *Mpo* mRNA expression was higher in the HFD group than that in the control group. Though the mRNA expression of *Tgfβ1* did not increase with LPS administration, a comparative analysis between the ND and HFD groups revealed higher levels in the HFD group. In contrast, superoxide dismutase 2 (*Sod2*) mRNA expression increased in the control group upon LPS administration, yet no such increase was noted in the HFD group following LPS administration.

### Effects of LPS administration on macrophage activation in the murine model of fatty liver

**Figure 6:**
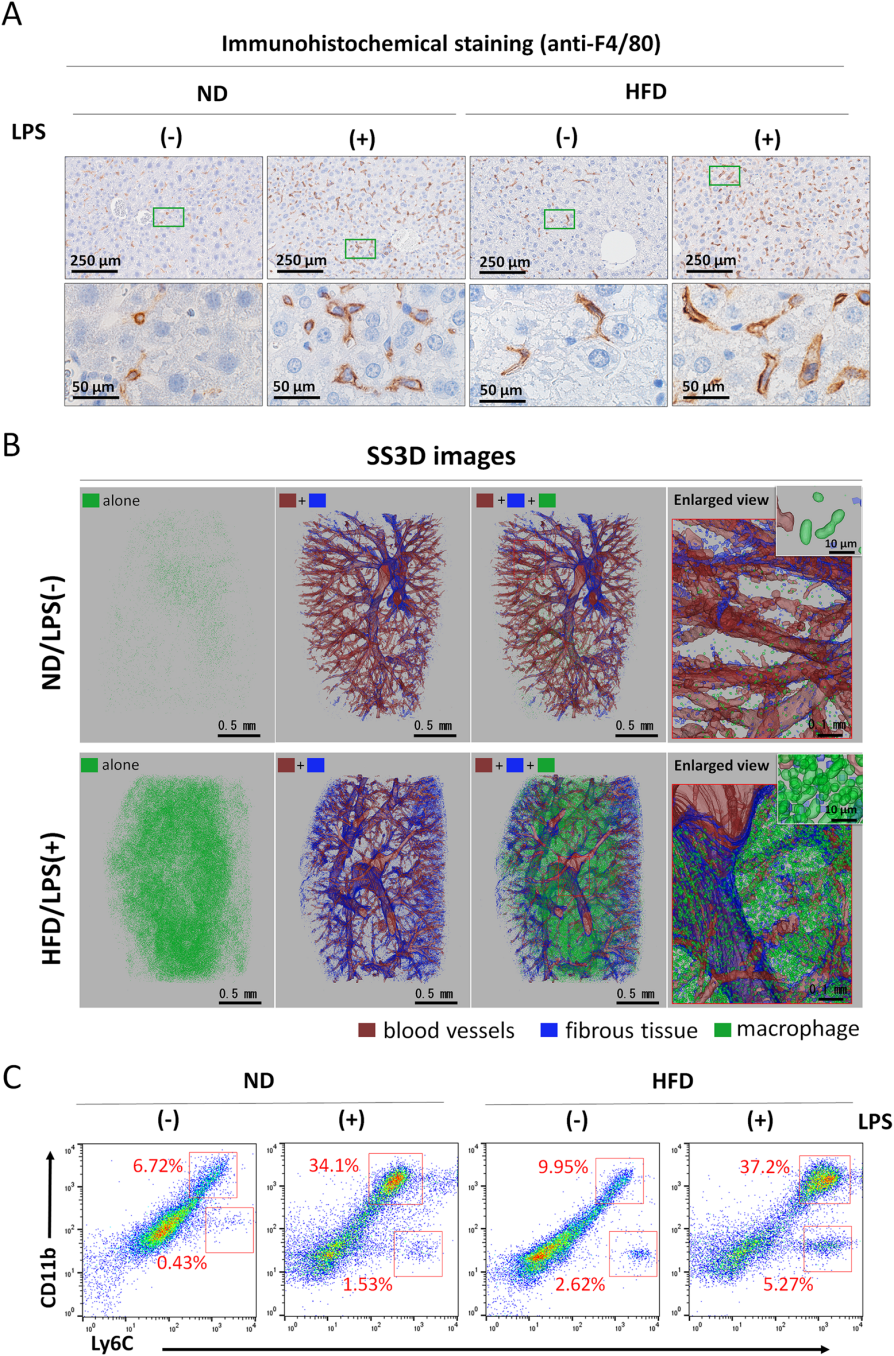
(**A**) Immunohistochemical staining of liver sections. Liver sections were prepared from each group, and then immunohistochemical staining using F4/80 antibody was performed. 3,3′-Diaminobenzidine (DAB) was used to detect the epidermal growth factor module-containing mucin-like hormone receptor-like 1 (F4/80) signals (brown). Nuclei stained with hematoxylin (blue). (**B**) Serial section-3-dimensional (SS3D) images of the murine livers with macrophage signals. Blood vessel (red), fibrotic tissues (blue), and macrophages (green) were detected and superimposed. Other details of the experiment are included in Materials and Methods. (**C**) Populations of lymphocyte antigen 6 complex, locus C1 (Ly6C)^+^ and/or integrin alpha M (CD11b)^+^ macrophages. To investigate the ratio of Ly6C^+^ and/or CD11b^+^ macrophages in total liver blood cells from mice treated as shown in Fig. 1A, fluorescence-activated cell sorting (FACS) analysis was performed with anti-CD11b fluorescein Isothiocyanate (FITC) and Gr-1 (Ly6C) Allophycocyanin (APC) antibodies. Typical data are shown. The other samples were similar in each group [(ND/LPS(−): n = 6; ND/LPS(+): n = 4; HDF/LPS(−): n = 6, HFD/LPS(+): n = 5]. HFD, high-fat diet; ND, normal diet; LPS, lipopolysaccharide.

As recent studies have shown that macrophages characterized by high F4/80 and Ly6C expression, which are activated in response to hepatocyte necrosis and inflammation, produce inflammatory cytokines such as TNF-α and IL-1β^33,34^, which exacerbates inflammation. In this study, the proportions of F4/80^+^ and Ly6C^+^ macrophages in the liver were analyzed. Immunohistochemical staining was conducted using an anti-F4/80 antibody (Fig. 6A). A significant increase in F4/80^+^ macrophages was observed in both ND and HFD groups following LPS administration. These macrophages were dispersed throughout the liver parenchyma, and comparison between the LPS-activated ND and HFD groups revealed no substantial difference in the elevated levels of F4/80^+^ macrophages. Similarly, the counts of F4/80^+^ macrophages in the ND and HFD groups without LPS administration showed no significant differences. Additionally, SS3D analysis was performed to compare the ND/LPS (−) and HFD/LPS (+) groups by extracting F4/80^+^ macrophage signals from immunohistochemical staining images (Fig. 6B). SS3D analysis indicated a pronounced increase in macrophages in the HFD/LPS (+) group compared with the ND/LPS (−) group. The smallest macrophage signals (green) were approximately 10–30 µm in diameter, which is consistent with the size of individual macrophages. No clusters of macrophage aggregation were detected; they were evenly distributed throughout the liver parenchyma.

Fluorescence-activated cell sorting (FACS) was utilized to assess the population of Ly6C^+^ macrophages (Fig. 6C). In addition, CD11b, a marker of macrophages, was used to investigate whether subpopulations were present in Ly6C^+^ macrophages. The proportion of Ly6C^+^ macrophages in the blood cell fraction increased with LPS administration in both ND and HFD groups, and this increase in the HFD group was slightly higher or equal to that of the ND group. However, in the absence of LPS, there was no discernible difference in the percentage of Ly6C^+^ macrophages between the ND and HFD groups. Moreover, the presence of CD11b^+^ and CD11b^–^ subpopulations was observed in the Ly6C^+^ macrophage population. The percentage of CD11b^–^ subpopulation increased the most in the LPS-administered HFD group compared with the ND/LPS(−) group; however, an increase in the CD11b^–^ subpopulation was also observed in the HFD group without LPS administration.

### Effect of co-culture with macrophages on HSC activation

**Figure 7:**
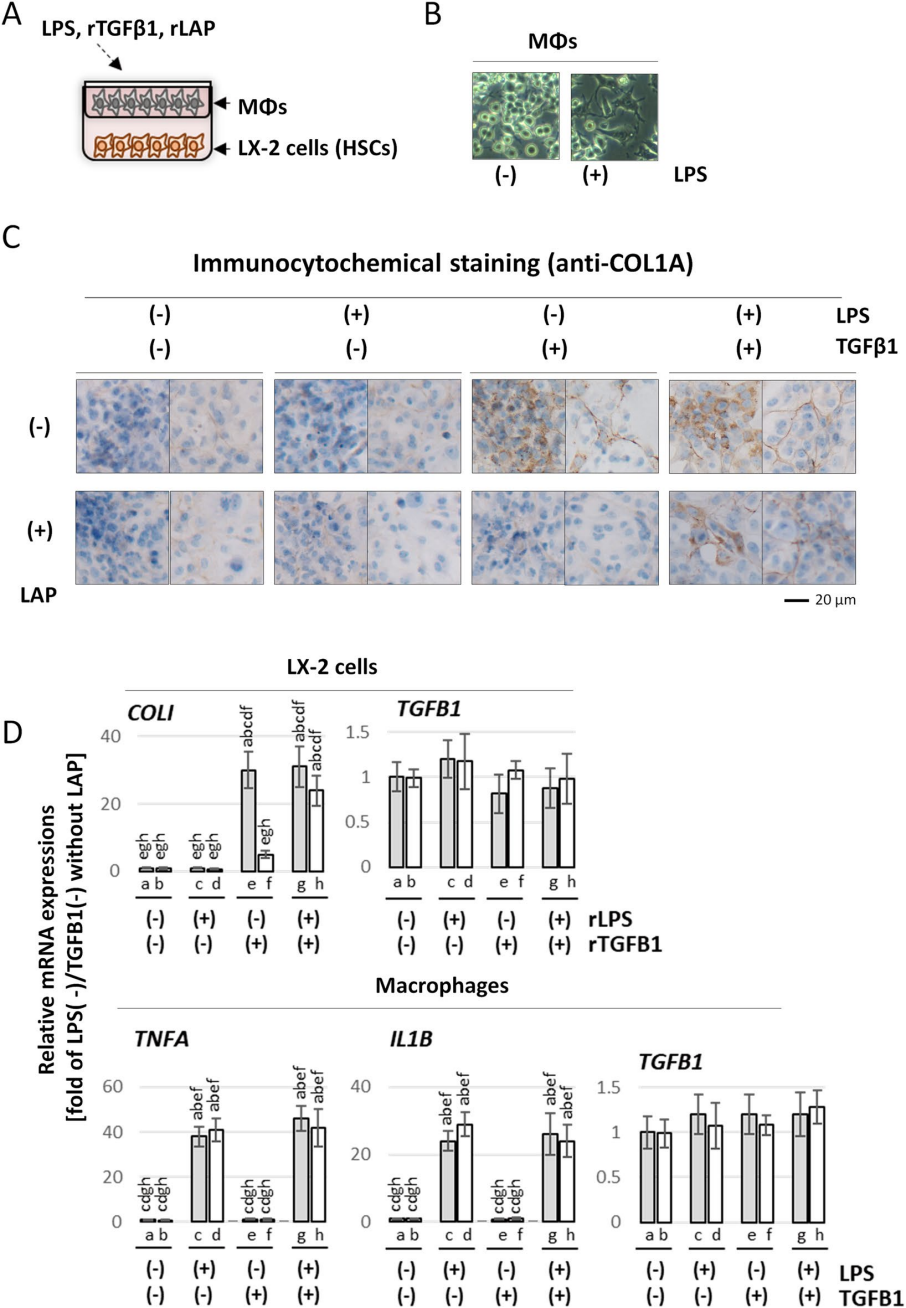
(**A**) Schematic image of co-culture. XL-2 cells, human hepatic stellate cell line, were co-cultured with primary human macrophages (MΦs) using a filter for 48 h in the presence or absence of lipopolysaccharides (LPS), recombinant human transforming growth factor beta 1 (TGFβ1) (active form) and/or latency associated peptide (LAP); subsequently, the underlying XL-2 cells were subjected to the following analysis. (**B**) Phase contrast images of macrophages cultured in the upper layer with or without LPS-administration alone. (**C**) Immunocytochemical staining images. Forty-eight after XL-2 cells were co-cultured with macrophages under the indicated each condition, immunocytochemical staining was performed using anti-collagen type 1a (COL1A1) antibody. 3,3′-Diaminobenzidine (DAB) was used to detect the COL1A1 signals (brown). Nuclei stained with hematoxylin (blue). (**D**) Expressions of COL1A1 and the inflammation-related genes. Real-time RT-PCR was performed to investigate the mRNA expression level of the indicated genes in the cells cultured under the same conditions as in (**B**). Quantitative data were presented as the mean ± SEM (n = 5). The letters above each bar in the graph indicate statistically significant differences (*P* < 0.05) compared to the corresponding sample group.

To validate our in vivo findings, an in vitro analysis was conducted. Macrophages and XL-2 cells, an HSC-like cell line, were co-cultured for 48 h with or without LPS (Fig. 7A) to explore the impact of the interaction between macrophages and HSCs on COL1A1 production. Given that Fig. 5 indicated an increase in TGF-β1 in the HFD group, conditions with and without TGF-β were analyzed to emulate HFD scenarios. Moreover, TGF-β1 is produced as a “latent” complex trapped in a prepeptide moiety called the latency-associated protein (LAP). The latent complex releases active TGF-β1 (25 kDa) upon stimulation by a stimulus^35^. Therefore, to investigate the state of TGF-β1, experiments with the addition of recombinant (r)LAP were performed. LPS administration prompted amoeboid-like morphological alterations in the macrophages cultured in upper layer (Fig. 7B). The results of immunohistochemical staining using anti-COL1A1 antibody of XL-2 cells cultured in the lower layer are shown in Fig. 7C. When rLAP was not administrated, a substantial production of COL1A1 in the XL-2 layer was observed with the rTGF-β1-administration, regardless of LPS administration. However, when rLAP was administered, remarkable COL1A1 production was only observed in the XL-2 layer co-administered with LPS and rTGF-β1. We further investigated *COL1A1* and *TGF-β1* mRNA expression levels in XL-2 cells cultured under the same conditions as shown in A (Fig. 7D). The results of *Col1a1* mRNA were consistent with the immunohistochemistry results. However, no significant difference in *TGF-β1* mRNA expression was observed under any culture conditions.

Subsequently, the mRNA expression of COL1A1 in XL-2 cells cultured under the same conditions as those in Fig. 7C was analyzed, yielding results akin to those depicted in Fig. 7C. Additionally, to corroborate the findings of Fig. 5, the mRNA expression levels of *Il-1β*, *Tnf-α*, and *Tgf-β1* in macrophages cultured under identical conditions were examined (Fig. 7D). *IL-1β* and *TNF-α* expression significantly increased following LPS administration regardless of the presence of rTGF-β1 and rLAP. On the contrary, no significant differences in *TGF-β1* expression were detected across all groups.

## Discussion

The Non-Alcoholic Fatty Liver Disease Activity Score (NAS) is utilized to grade the severity of NASH, a liver condition characterized by inflammation and fat accumulation^42^. The NAS is based on three histological components: steatosis (fat accumulation in liver cells), lobular inflammation, and hepatocellular ballooning. Each component is scored from 0 to 3 based on the severity of involvement, with higher scores indicating more severe manifestations. Steatosis is graded based on the percentage of hepatocytes affected, whereas lobular inflammation is assessed by the number of inflammatory foci per field of view. Hepatocellular ballooning reflects the presence of enlarged and rounded hepatocytes indicative of cellular injury. The NAS is calculated by summing the individual scores of these components, with a total score ranging from 0 to 8. An NAS of 5 or higher is typically indicative of NASH. In our HFD/LPS(+) mouse model, notable hepatocellular ballooning was observed, but there were no notable inflammatory foci and steatosis to be scored. On the basis of NAS, this finding suggests that our model is not perfect. In this study, in order to minimize discomfort to the mice, LPS administration was performed just once; furthermore, the mice were housed for a short period (48 h) thereafter. To better mimic human NASH, it may be necessary to conduct experiments involving multiple administrations over a longer duration to induce chronic inflammation. However, interpretations of NAS may vary, and consideration of additional factors such as fibrosis stage is essential for a comprehensive assessment of NASH severity. This study highlights the applicability of the SS3D method for visualizing and quantifying fibrotic tissue across the entire liver in mice. The SS3D images, along with 2D images of sectioned specimens, were effective in detecting increased fibrotic tissue around sinusoidal capillaries and blood vessels. Moreover, the SS3D method facilitated the visualization and quantitative analysis of macrophage localization in the liver. Notably, the size of the macrophages corresponded closely to a single signal indicating macrophages in the SS3D images, thus validating the usefulness of SS3D analysis in assessing fibrosis status comprehensively. Our observations revealed that macrophages were dispersed throughout the liver parenchyma. Therefore, the inflammatory response leading to fibrosis induced by HFD and LPS is unlikely to be confined to a specific location within the liver.

In this study, we observed an increase in *Tnfα*, *Il1β*, and *Mpo* mRNA expression levels following LPS administration, with a more pronounced elevation in the HFD group compared with the ND group. These genes are known to induce or exacerbate inflammation^36^. In contrast, *Sod2* mRNA expression, which was elevated in the control group after LPS administration, showed a suppressed increase in the HFD group following LPS treatment. SOD2 is an enzyme that mitigates ROS and possesses anti-inflammatory properties. The inhibition of *Sod2* mRNA expression is associated with the aggravation of inflammation^37^. Therefore, modulating the expression of these inflammation-related genes is likely a key factor in the exacerbation of fibrosis in fatty livers due to LPS. On a different note, the mRNA expression of *Tgf-β1* did not increase with LPS administration. However, a comparison between the ND and HFD groups revealed higher levels of *Tgf-β1* in the HFD group. Connective tissue growth factor (*Ctgf*), which operates downstream of the TGF-β signal, is strongly linked to the progression of liver fibrosis^38,39^. In a rat fibrosis model, the administration of *Ctgf* siRNA has been reported to inhibit fibrosis progression. Thus, it can be inferred that Tgf-β1, which is associated with fatty liver, contributes to fibrosis.

Macrophages significantly contributed to the elevated expression of inflammatory factors in the HFD group. Inflammatory mediators, produced by DAMPs, attract macrophages to sites of inflammation, where they accumulate^40^. Once activated, macrophages secrete factors that exacerbate inflammation. Specifically, in the liver, F4/80^+^ Ly6C^+^ macrophages are known to produce inflammatory cytokines such as TNF-α and IL-1β^33,34^. These cytokines activate HSCs, leading to collagen fiber production, which is foundational to fibrosis. The obtained immunohistochemical images revealed that LPS administration increased the number of F4/80^+^ macrophages in both the ND and HFD groups. Similarly, FACS analysis showed that the ratio of Ly6C^+^ macrophages increased with LPS administration in both ND and HFD groups. Thus, the majority of F4/80^+^ macrophages that increased with LPS administration are considered to be Ly6C^+^ macrophages, which is consistent with previous reports. Therefore, these macrophages must be responsible for the increased mRNA expression of *Tnf-α* and *Il-1β* detected in this study. Moreover, CD11b^–^ and CD11b^+^ subpopulations were found to be present in Ly6C^+^ macrophages. The reason for the division of these macrophages into two subpopulations is currently unknown, but it may play an important role in fibrosis of the liver caused by the synergistic effect of HFD and LPS. Recent research has revealed a diverse array of macrophage types in NASH, including yolk sac-derived Kupffer cells and bone marrow-derived TIM4-positive cells that differentiate from Ly6C^+^ macrophages^41^. Furthermore, single-cell analysis of human fibrotic liver has led to the classification of macrophages into seven distinct clusters^41^. These findings suggest the potential for subpopulations among macrophages that increase in number due to LPS administration and HFD.

Finally, building upon these findings, we further explored the interaction between macrophages and HSCs and the signaling mechanisms underlying fibrous tissue formation. This was accomplished by co-culturing macrophages with XL-2 cells. The morphology of the macrophages in the upper layer changed following the addition of LPS. Moreover, LPS administration led to an increased expression of TNF-α and IL-1β in the macrophages, indicating their activation in this experimental setup. Co-culture experiments revealed that rTGF-β1 alone activated XL-2 cells to produce COL1A1, which was inhibited by LAP. Interestingly, this inhibition of rTGF-β1-induced XL-2 cell activation by LAP was rescued by co-culture with LPS-administered macrophages. These results suggest that activated macrophages convert latent TGF-β1, a complex with LAP, into the active form, which activates HSCs. This can successfully explain the results of in vivo experiments showing that HFD increased *Tgf-β1* mRNA expression in the liver. However, a notable increase in fibrosis in the liver was observed only when LPS was administered, i.e., when the number of macrophages increased. It has been reported that kallikrein activates TGF-β1 and that TNF-α increases urokinase-type plasminogen activator (uPA) in HSCs, which in turn activates kallikrein^42^. This supports the results of this study. However, one question arises: Which cells produce the latent form of TGF-β1, which is activated in the liver by HFD. Activated HSCs produce TGF-β1, which acts in an autocrine manner. Some types of macrophages also produce TGF-β1. However, in our co-culture experiments, the expression levels of *TGF-β1* in these cells did not change under any culture conditions; even in in vivo experiments, HFD induced an increase in *Tgf-β1* mRNA expression without LPS administration. According to previous reports, hepatocytes damaged by fat accumulation release DAMPs, which stimulate various types of cells^6–11, 15–18^. In addition, our FACS analysis showed that there is an LPS-independent macrophage subpopulation (Ly6C^+^ CD11b^–^) that is increased by HFD. Studies on the relationship between these cells and DAMPs may reveal the cells responsible for the HFD-induced increase in TGF-β1.

In conclusion, our SS3D method has proven effective in creating 3D images of fibrotic liver tissue. This technique offers a broad field of view and high resolution, which are anticipated to significantly contribute to future research on NASH and various other diseases. Regarding the mechanism underlying the progression of fibrosis in fatty liver, it has been suggested that fatty liver contains target cells for DAMPs other than HSCs and LPS-activated macrophages, which produce TGF-β1. This TGF-β1 then activated by LPS-associated macrophages that produce inflammatory cytokines such as TNF-α and IL-1β, which results in the activation of HSCs to express COL1A1. Further investigation into the mechanism of DAMPs in fatty liver is essential to enhance our understanding of the pathogenesis of NASH.

### Supplementary Information

Supplementary Legends.

Supplementary Information 2.

Supplementary Information 3.

## Data Availability

All data generated or analyzed during this study are included in this published article and its supplementary information files.
