# Supplementary material for: Assessing the combined impact of fatty liver-induced TGF-β1 and LPS-activated macrophages in fibrosis through a novel 3D serial section methodology
Source: Sci Rep. 2024 May 18;14:11404. doi: 10.1038/s41598-024-60845-6 (PMC11102459; doi:10.1038/s41598-024-60845-6)
Supplement: Supplementary file 1 — Supplementary Legends. [file 41598_2024_60845_MOESM1_ESM.docx]

**Supplemental data legends**

**Supplemental data 1**. Schematic diagram of serial mounting of paraffin sections on glass slides. Fixed samples were dehydrated and embedded in paraffin. Paraffin-embedded blocks were trimmed and sectioned at 5 µm thickness using a microtome. Every fifth section was mounted on a glass slide and dried, until there were three sections per slide. Different stains were applied to sections in each category.
