# Supplementary figures and images for: Assessing the combined impact of fatty liver-induced TGF-β1 and LPS-activated macrophages in fibrosis through a novel 3D serial section methodology
Source: Sci Rep. 2024 May 18;14:11404. doi: 10.1038/s41598-024-60845-6 (PMC11102459; doi:10.1038/s41598-024-60845-6)

Sequential number of sections

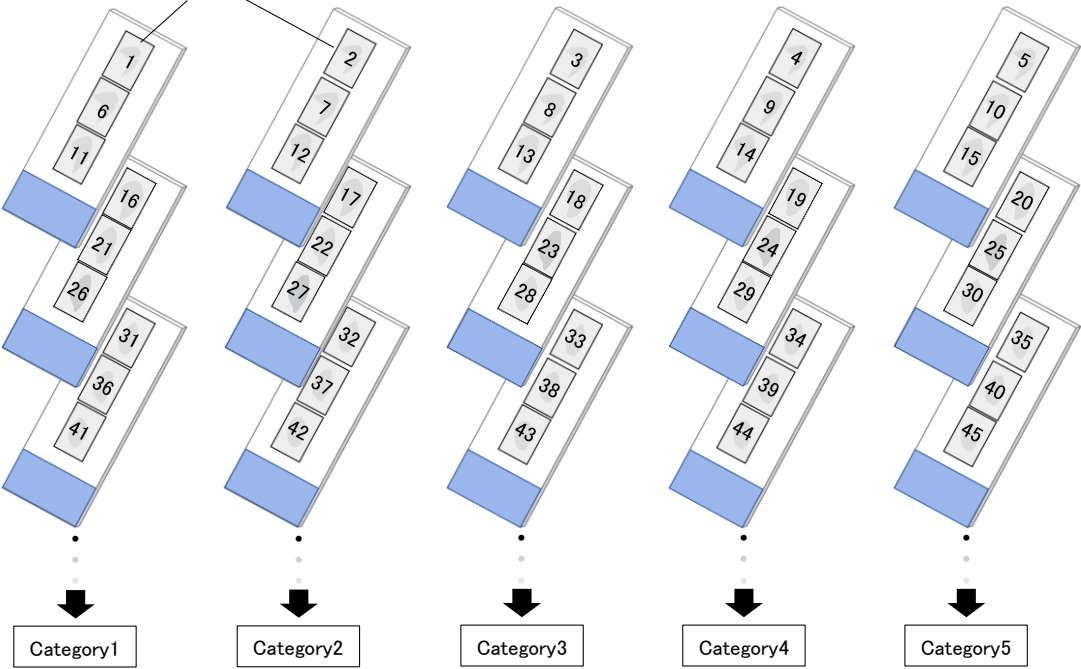

Supplement: Supplementary file 2 — Supplementary Information 2. [file 41598_2024_60845_MOESM2_ESM.pdf]
