# Supplementary material for: Assessing the combined impact of fatty liver-induced TGF-β1 and LPS-activated macrophages in fibrosis through a novel 3D serial section methodology
Source: Sci Rep. 2024 May 18;14:11404. doi: 10.1038/s41598-024-60845-6 (PMC11102459; doi:10.1038/s41598-024-60845-6)
Supplement: Supplementary file 3 — Supplementary Information 3. [file 41598_2024_60845_MOESM3_ESM.docx]

| **Supplemental data 2. The sequence of oligonucleotide primers used for real-time RT-PCR** | |
| --- | --- |
| **Target** | **Sequence** |
| **mouse** |  |
| *Gapdh* | 5’-GGGTTCCTATAAATACGGACTGC-3’ 5’-CCATTTTGTCTACGGGACGA-3’ |
| *Il1b* | 5’-TCTTCCTAAAGTATGGGCTGGA-3’ 5’-AAAGGGAGCTCCTTAACATGC-3’ |
| *Il-5* | 5’-ACATTGACCGCCAAAAAGAG-3’ 5’-ATCCAGGAACTGCCTCGTC-3’ |
| *Il6* | 5’-CGCTATGAAGTTCCTCTCTGC-3’ 5’-TTGGGAGTGGTATCCTCTGTG-3’ |
| *Il13* | 5’-GTGTCTCTCCCTCTGACCCTTA-3’ 5’-GGGGAGTCTGGTCTTGTGTG-3’ |
| *Il17a* | 5’-CAGGGAGAGCTTCATCTGTGT-3’ 5’-GCTGAGCTTTGAGGGATGAT-3’ |
| *Il21* | 5’-GACATTCATCATTGACCTCGTG-3’ 5’-TCACAGGAAGGGCATTTAGC-3’ |
| *Il22* | 5’-TGACGACCAGAACATCCAGA-3’ 5’-AATCGCCTTGATCTCTCCAC-3’ |
| *Il23a* | 5’-TCCCTACTAGGACTCAGCCAAC-3’ 5’-AGAACTCAGGCTGGGCATC-3’ |
| *Irf1* | 5’-GAAGAATATGGACCCCGTCAT-3’ 5’-TATGGTGCACAAGGAATAGCC-3’ |
| *Mpo* | 5’-CTGAATCCTCGATGGAATGG-3’ 5’-CCATGGCCCCTACAATCTT-3’ |
| *Sod2* | 5’-TGGACAAACCTGAGCCCTAA-3’ 5’-GACCCAAAGTCACGCTTGATA-3’ |
| *S100a4* | 5’-TGCATTCCAGAAGGTGATGA-3’ 5’-CTCCTGGAAGTCAACTTCATTGT-3’ |
| *S100a6* | 5’-ACTCTGGCAAGGAAGGTGAC-3’ 5’-CAATGGTGAGCTCCTTCTGG-3’ |
| *S100a8* | 5’-TGCGATGGTGATAAAAGTGG-3’ 5’-GGCCAGAAGCTCTGCTACTC-3’ |
| *S100a9* | 5’-AATGGTGGAAGCACAGTTGG-3’ 5’-CTGATTGTCCTGGTGCTCAG-3’ |
| *S100a10* | 5’-CCTCTGGCTGTGGACAAAAT-3’ 5’-AAGCCCACTTTGCCATCTC-3’ |
| *S100a11* | 5’-CCCAGAAGCGAATCTAATCCT-3’ 5’-AGGCGTGGGATACATGTTGT-3’ |
| *Tgfb1* | 5’-TCAGACATTCGGGAAGCAGT-3’ 5’-ACGCCAGGAATTGTTGCTAT-3’ |
| *Tgfb2* | 5’-TGGAGTTCAGACACTCAACACA-3’ 5’-AAGCTTCGGGATTTATGGTGT-3’ |
| *Tgfb3* | 5’-TGGCTGTCTTTCGATGTCAC-3’ 5’-GCTGATTTCCAGACCCAAGT-3’ |
| *Tnfa* | 5’-TCTTCTCATTCCTGCTTGTGG-3’ 5’-CACCCCGAAGTTCAGTAGACA-3’ |
|  |  |
| **human** |  |
| *GAPDH* | 5’-TGTGGGCATCAATGGATTTGG-3’ 5’-ACACCATGTATTCCGGGTC-3’ |
| *IL1B* | 5’-AGGGCCAAGACGAAGACATC-3’ 5’-CAGATCACGTCATCGCAC-3’ |
| *TGFB1* | 5’-GCCAGATCCTGTCCAAGC-3’ 5’-GTGGGTTTCCACCATTAGC-3’ |
| *TNFA* | 5’-ATTCTGAGCAAAATAGCCAGC-3’ 5’-GGCTTCCTTCTTGTTGTGTG-3’ |
| *COL1A1* | 5’-AGGGCCAAGACGAAGACATC-3’ 5’-AGATCACGTCATCGCACAAC-3’ |
